# Fibrous flexor sheath ganglion and trigger thumb in a 14-year-old female

**DOI:** 10.4103/0970-0358.53029

**Published:** 2009

**Authors:** S. S. Suresh, V. Rani

**Affiliations:** Ibri Regional Referral Hospital, PO Box 46, Ibri 516, Sultanate of Oman

**Figure 1:**
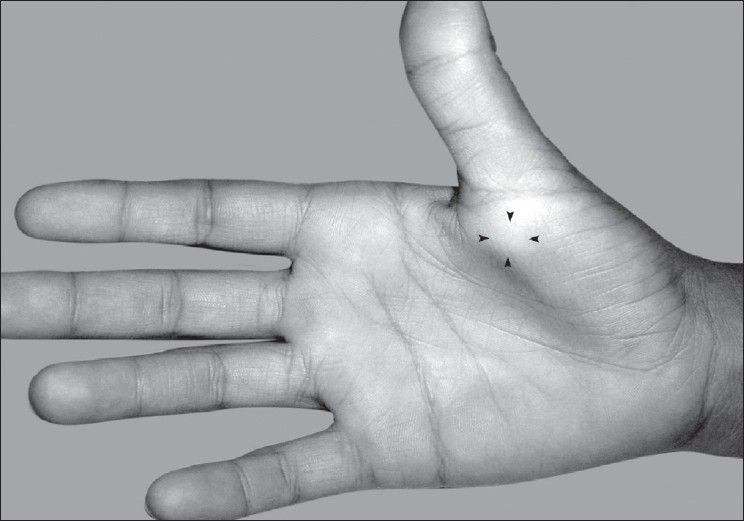
Swelling over the A1 pulley of right thumb (black arrow heads)

**Figure 2:**
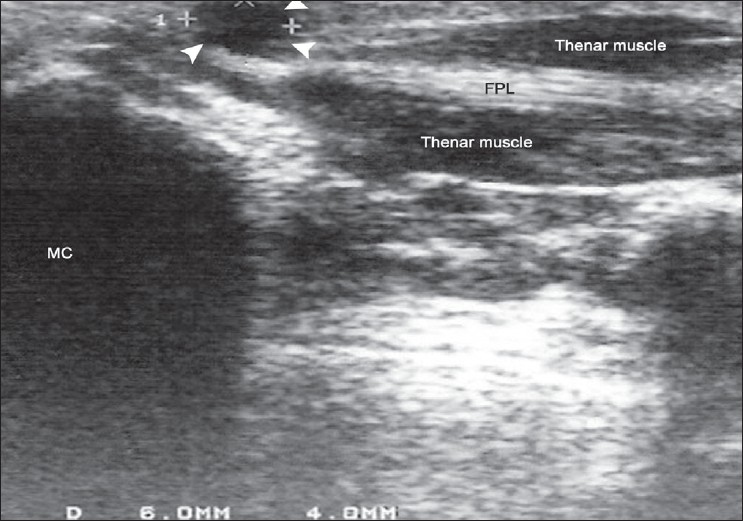
An USS of the right thumb, showing the relation of the ganglion (white arrow heads), MC-metacarpal

**Figure 3:**
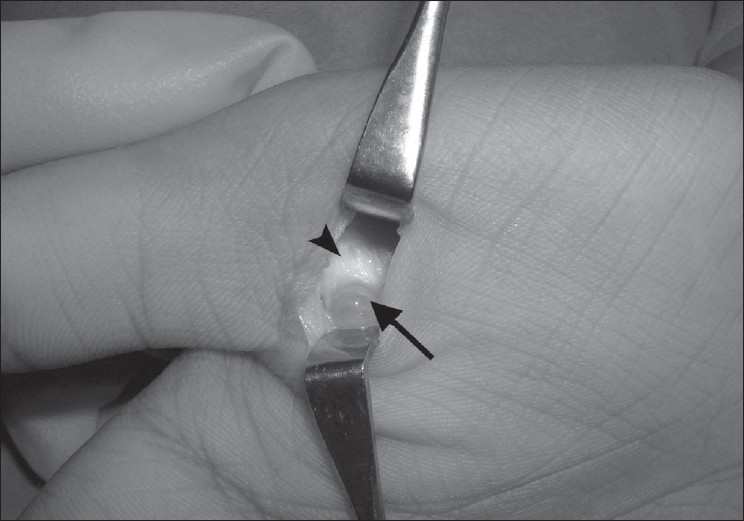
A ganglion (black arrow) arising from the flexor tendon sheath (black arrow head)

**Figure 4:**
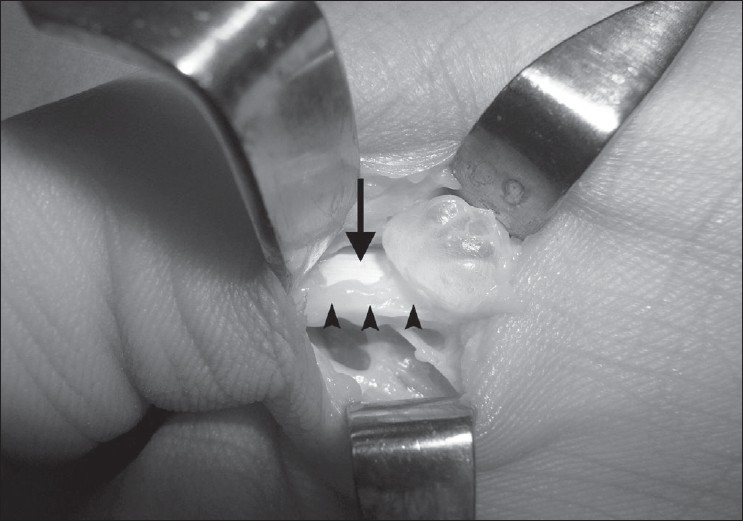
Intra operative photograph, showing ganglion, FPL (black arrow), and synovial hypertrophy (black arrow heads)

Sir,

A 14-year-old female presented with a painful swelling of the right thumb of over 1 year duration. Her initial complaint was pain on holding a pen, but subsequently she noticed progressive triggering of the thumb. A small mass less than 10 mm could be felt over the A1 pulley [Figure 1]. The mass was not mobile while on extension of the thumb, but slight mobility could be appreciated while the thumb was flexed. She could flex the thumb fully, but during active extension the thumb became locked in a flexed position. There was no neurological deficit in the hand. An ultrasound scan (USS) showed a cystic round mass over the flexor pollicis longus (FPL) tendon measuring 6 × 8 mm attached to the volar aspect of the flexor tendon sheath [Figure 2]. A dynamic USS showed trivial movement of the cyst with the FPL. The patient underwent surgery with a Bier's block through a transverse incision over the flexor crease of the thumb. The mass was found arising from the tendon sheath of the FPL at the level of the A1 pulley. A pearl shaped globular mass 6 × 7 mm in size was excised. The tendon sheath was found hypertrophied [Figure 3 and 4]. The A1 pulley was released and the hypertrophic synovial tissue was excised. A bisected mass yielded gelatinous material consistent with ganglion cyst. Free excursion of the tendon was noted at the end of the procedure. At six 6-month follow-up visit, the patient was symptom free and had no triggering.

Trigger finger is uncommon in children and usually presents as congenital trigger thumb in early infancy. Tenosynovitis primarily involving the A1 pulley is the most common cause of trigger finger.[1] Any cause resulting in a mismatch between the tendon size and the space available for its gliding can lead to triggering. Soft tissue tumors are reported to cause triggering due to compression of the tendon from outside or reactive synovial proliferation. Reported causes of triggering of the fingers include enlarged radial sesamoid, partial laceration of the flexor tendon, calcifying aponeurotic fibroma, localized amyloid deposit, giant cell tumor of the tendon sheath, soft tissue chondroma, fibroma of the tendon sheath, foreign body granuloma, intra tendinous granulation tissue, inter tendinous connections between the flexor tendons, flexor tendon sheath ganglia, and tenosynovial osteochondroma.[1,2]

Ganglion as a cause of triggering was mentioned by two authors[2,3] and Jebson analyzed flexor tendon ganglions and found a correlation. Jebson has mentioned that he releases the A1 pulley if there is an associated trigger finger in association with flexor tendon sheath ganglia.[3] In their series, nearly half of the flexor sheath ganglions arose from the A1 pulley of which seven had triggering. Triggering was observed in nearly one fourth of the patients in their series. A possible mechanism of triggering could be the constrictive effect of the tumor on the tendon sheath or associated intra luminal synovial hypertrophy.

Ganglion is the most common soft tissue tumor of the hand. Approximately 10% of the ganglia arise from the A1 pulley and are called fibrous flexor sheath ganglia.[3] Flexor tendon sheath ganglions are called pearl or seed ganglions due to their typical shape.[4]

Ganglion formation, in addition to narrowing and thickening of the sheath at the level of the A1 pulley, has been suggested as a cause of triggering.[2]

Conservative treatment is unsuccessful when there is a combination of flexor sh eath ganglion and trigger digit and excision is the treatment of choice.

In our patient, in addition to the pearl ganglion, there was a synovial hypertrophy resulting in compromised gliding of the FPL.
